# Surgical Treatment of Spinal Fracture in a Patient With Ankylosing Spondylitis: An Opportunity To Correct Spinal Deformity Simultaneously With Fracture Fixation?

**DOI:** 10.7759/cureus.21936

**Published:** 2022-02-05

**Authors:** Wiktor Urbański, Rafal Zaluski

**Affiliations:** 1 Department of Neurosurgery, Wroclaw Medical University, Wroclaw, POL

**Keywords:** spinal surgery, pedicle subtraction osteotomy, hyperkyphosis, cervical fracture, ankylosing spondylitis

## Abstract

The objective of this case report is to describe the substantial sagittal correction of spinal hyperkyphosis alongside fracture fixation. In advanced ankylosing spondylitis (AS), the spine is usually fused, hyperkyphotic, and due to deformity, as well as improper bone remodeling, predisposed to fractures. These fractures, mostly unstable, require surgical treatment. The authors present fracture management with concomitant deformity correction at the fracture site and pedicle subtraction osteotomy (PSO) below the fracture, showing the benefits of performing the procedures with the patient in a sitting position.

A 58-year-old male with AS was diagnosed with a fracture of C6 and referred to the department of neurosurgery, Wroclaw University Hospital. For the last week, he had complained of worsening neck pain and exacerbation of spinal kyphosis, with no neurological deficits. The patient had a fully fused spine, significant hyperkyphosis prior to the injury, and a fracture with an additionally exacerbated deformity. The patient was offered operative treatment - spinal fusion and fracture reduction with hyperkyphosis correction. The procedure consisted of 1) partial, mostly closed correction at the fracture site, 2) PSO of C7 and C2-T3 pedicular fixation and fusion while sitting in the posterior approach. To enable closed reduction at the fracture site and avoid difficulties with positioning a prone patient with very severe hyperkyphosis and an unstable spine, the authors performed surgical procedures with the patient in a sitting position.

The authors obtained significant correction during the procedure by 74^0^, from 53.4^0^ of kyphosis to 24.3^0^ of lordosis measured between C2 and T1. The patient had several complications (transient weakness of the upper limb, pleural effusion, and delayed wound healing); however, all resolved and the patient was discharged within two weeks post the operation.

In patients with spinal hyperkyphosis with AS who sustain spinal fractures requiring operative treatment, it is worth considering simultaneous correction of the spinal deformity during surgical management of the fracture.

## Introduction

Advanced cases of ankylosing spondylitis (AS) are usually associated with restriction of mobility and debilitating deformity of the spine and other joints. There is an elevated fracture risk in AS due to pathological spinal remodeling and osteoporosis [1-2]. Ectopic bone forms with unusual osteoproliferative processes leading to a ligamentous ossification progressively bridging the whole spine and simultaneously develops osteopenia - in part resulting from a stress shielding of the cancellous vertebral parts [3]. The fractures are usually associated with low-energy trauma. Patients with AS have often a disturbed line of vision and they are prone to falls; 40% of fractures are caused by simple ground-level falls [4]. Due to deformity with altered spinal balance, the fracture may have a mechanism of fatigue fracture. It is often an expression of patients’ effort to properly align the head and/or compensate spinal balance. The fractures are frequently recognized with delay, which increases the risk of secondary neurologic injury due to high instability and potential displacement; the spine acts as a long bone. The timing may also add to deformity development or, more likely, a deterioration of the present one. The diagnostic issues are driven by the often uncertain mechanism of trauma (low-energy) and are asymptomatic. Thus, the sooner the treatment begins the better.

The treatment strategies for patients with AS have to be different from patients with non-rigid spines. Conservative treatment is rarely an option and is chosen only in uncommon cases of stable fractures [5]; however, nonoperative treatment is associated with the risks of healing problems and pseudarthrosis [6]. Longer immobilization and bed rest may result in general medical complications (pulmonary, urinary infections, thromboembolism, etc.). Moreover, the selection and fitting of the proper external immobilization may be extremely difficult due to deformity. Since spinal fractures in AS are usually highly unstable, with three columns involved, and the risk of primary and secondary spinal cord injury, the majority of patients require surgery [6].

The surgery itself presents several issues. Besides the fact that AS patients commonly present comorbidities, the number of complications related to surgery remains high [5-7]. Especially, these patients require long constructs, extensive surgery with decompression, and, at least, realignment of the spine, if not deformity correction [7].

## Case presentation

**Figure 1:**
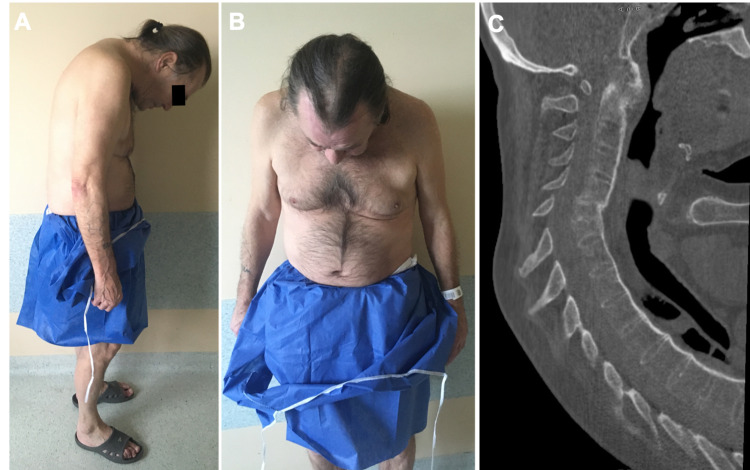
Patient with AS and the chin-on-chest deformity exacerbated by a C5 fracture A) and B) clinical images. B) CT sagittal MPR showing completely fused spine, displaced fracture, cervical hyperkyphosis AS: ankylosing spondylitis

A 58-year-old male was treated for ankylosing spondylitis for many years with anti-inflammatory drugs (steroids and non-steroidal anti-inflammatory drugs (NSAIDs)). Due to his medical history, he has never been offered biologic therapy. He was a patient with multiple comorbidities, with a history of myocardial infarction treated with a percutaneous coronary intervention (PCI) of the left anterior descending (LAD) artery and insertion of a drug-eluting stent (DES) (PCI LAD + DES) seven years ago, left-sided pleural empyema 20 years ago, depression - on psychiatric medication, heavy smoker (up to two packets a day). He was referred to the department of rheumatology, Wroclaw University Hospital, due to an exacerbation of neck pain and progression of deformity, leading to the “chin on chest” deformity and problems with swallowing for the last week (Figures 1A-1B).

The patient was neurologically intact, with painful neck and swallowing problems gradually deteriorating for the last week at presentation and chin-on-chest deformity with two fingers between chest and chin. The patient’s pre-existing spinal deformity along with newly developed deformity due to fracture has not allowed for any external immobilization. The CT scan revealed a C6 fracture - unstable, with involvement of all three columns, subluxation, exacerbation of hyperkyphosis of the cervical spine, and fully fused spine (Figure 1C).

The patient was offered operative treatment, which consisted of spinal fixation and fracture reduction with the option of kyphotic deformity correction. After careful analysis, the decision was made to fix the fracture and correct the pre-existing kyphosis from the posterior approach at the same sitting - partial correction at the fracture site but also pedicle subtraction osteotomy (PSO) of C7 and C2-T3 fixation.

**Figure 2:**
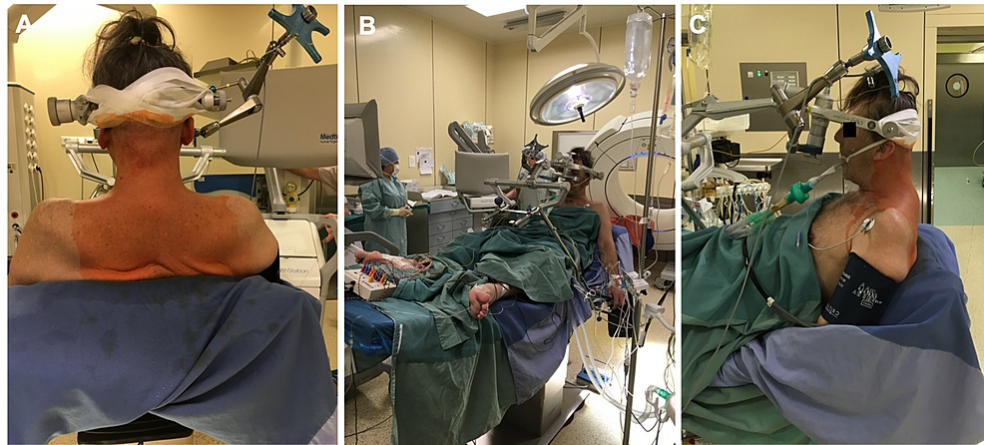
Positioning of the patient on the operating table in a sitting position The arm of the Mayfield clamp was mounted on the table with the aid of gravity, which allowed for gradual reduction under carefully controlled neuromonitoring.

**Figure 3:**
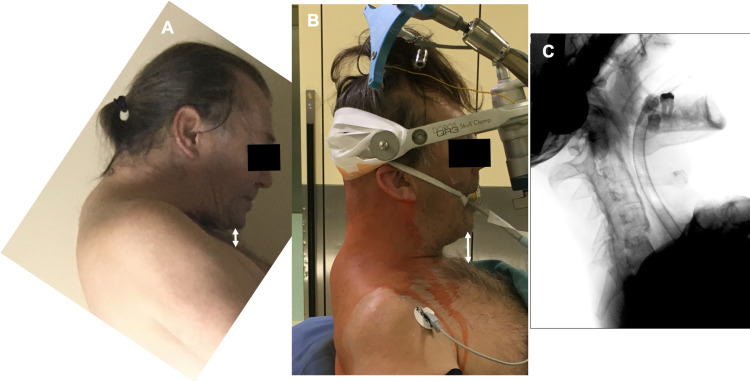
A) Preoperative chin-chest distance; B) Chin-chest distance increased after patient positioning and gentle closed reduction; C) Fluoroscopy after closed reduction

After anesthesia and installation of neuromonitoring, the Mayfield clamp was attached to the patient’s head and the patient was positioned in a sitting position for the operation (Figure 2). With the patient sitting, with an immobilized head, mild traction was applied - gravitation plus gentle manipulating of the clamp arm and operating table under careful neuromonitoring, which resulted in gradual kyphosis correction (Figure 3).

**Figure 4:**
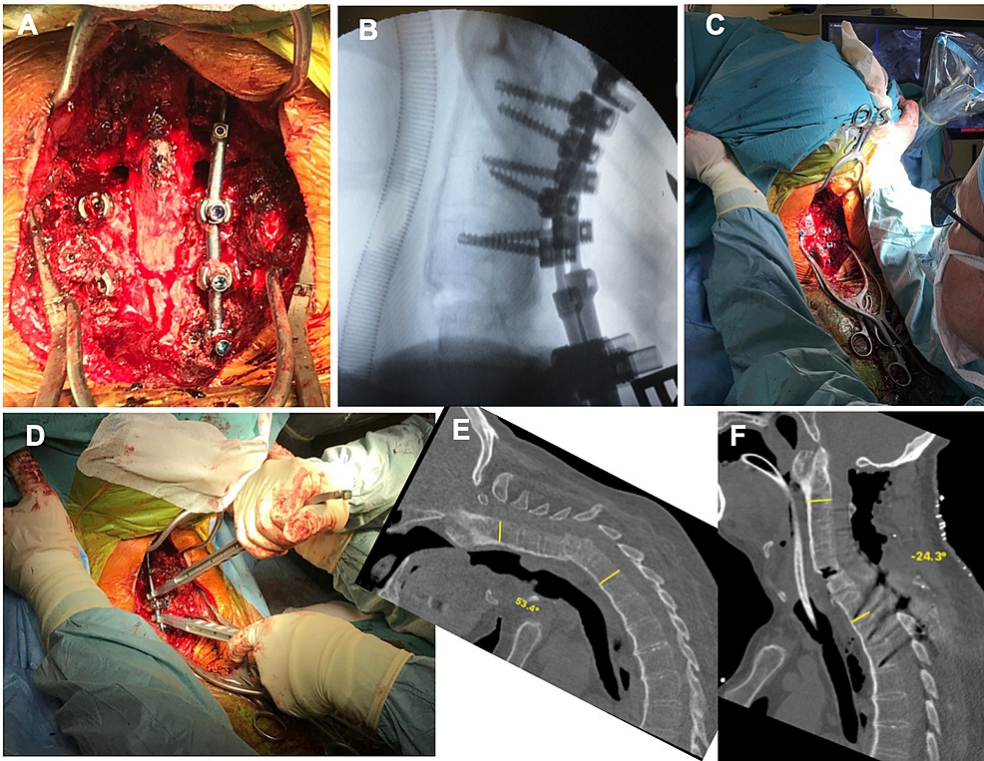
A) Wide laminectomy with C7 PSO performed and C7 and C8 roots decompressed before closure; B) Image from intraoperative fluoroscopy after osteotomy closure. Opening of the anterior column at the fracture site attained during primary closed reduction; C) Closing of the osteotomy site along with head repositioning. D) in situ rod bending E) preop CT scan with 53.4 of cervical kyphosis and F) postop CT scans showing 24.3 degrees of lordosis.

A skin midline incision was made and a standard subperiosteal dissection was performed. The reference frame for navigation purposes was mounted on the spinous process. An O-arm scan was performed. With the aid of navigation (S8, Medtronic Plc, Dublin, Ireland) based on the 3D image, pedicular screws were introduced from C2 to T3 bilaterally. After wide C5-T2 laminectomy, pedicle subtraction osteotomy was performed at the C7 level. Before osteotomy began, transition thoracocervical rods 5.5/3.5 were inserted and maintained throughout the procedure continuously and changed only from left to right. Once PSO bone removal was completed, the osteotomy was closed by compression of both rods, alongside gentle manipulating of the clamp’s arm and operating table to obtain sagittal correction. Additionally, in-situ rod bending was done for final realignment (Figure 4). The whole procedure was done under meticulous neuromonitoring; no significant changes in traces were noted at any stage. The whole procedure, from the positioning of the patient to wound closure and removal of the head clamp took 7 h 35 min.

Postoperatively, patients had prolonged respiratory support due to ventilation problems for two days. He presented with minor weakness of right-sided C5 and C6 (3/5), which resolved completely in two months. He developed pleural effusion and subsequently underwent drainage of the pleural cavity seven days after the operation and was maintained for four days. The wound healing was prolonged but finally fully healed in 28 days from the operation.

**Figure 5:**
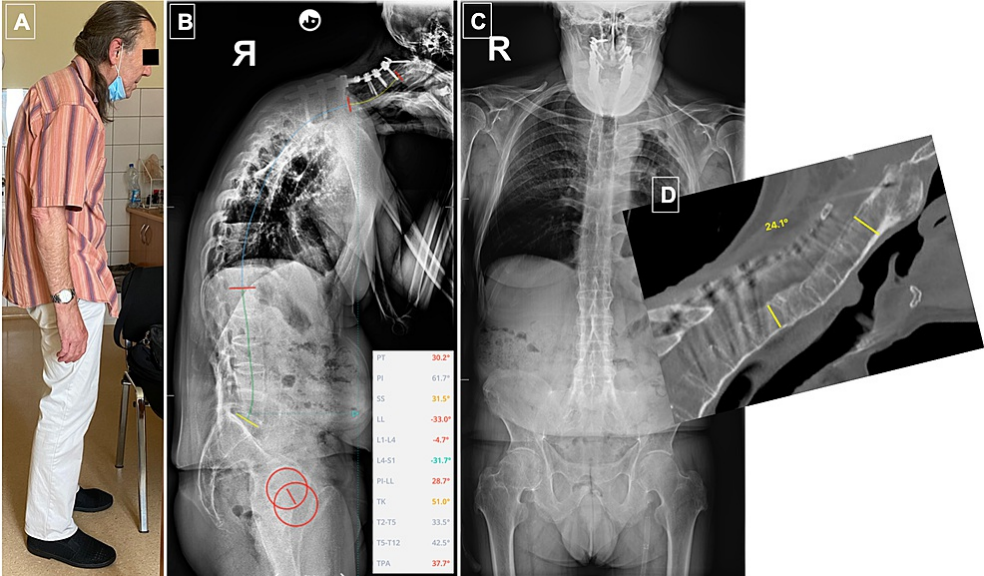
Six months after surgery A) Clinical picture showing satisfactory head position; B) Full spine anteroposterior (AP) and lateral X-rays; C) Significantly unbalanced spine sagittally, significantly positive sagittal vertical alignment (SVA); D) CT scan showing fusion of the anterior column of the spine

At the one-year follow-up, no further complications occurred. We offered the patient continuation of treatment and PSO of the lumbar spine. However, he refused, claiming that he is fully satisfied and did not wish for further operations (Figure 5).

## Discussion

In the presented case, surgeons performed fracture fixation and local kyphosis correction. The authors assume that the amount of correction available at the fracture site by opening the anterior column would be insufficient to realign the spine. Therefore, after close reduction and spinal fixation, pedicle subtraction osteotomy of C7 was performed. These two stages provided 78 degrees of correction in the sagittal plane.

In current literature, the concept of deformity correction in the cervical spine at the time of fracture treatment is known. Usually, the reduction is based on traction and gradual correction, but the reduction may be also achieved with intraoperative maneuvers (translation, compression, and in-situ rod bending). Manipulations at the site of the fracture result in the opening of the anterior column, thus an anterior column gap is formed and it may require to be filled in an additional anterior approach (if anatomical circumstances allow for anterior approach) [8-9]. In the presented case, the gap had appeared but was not large, and it healed fully in the six-month follow-up (Figure 5).

In the severely deformed spine in AS, difficulties with positioning the patient on the operating table are not uncommon. It gets even more complex if the spine is additionally unstable. In the presented case, the authors have chosen a sitting position in order to avoid struggling to position the patient and concomitantly obtain closed correction (traction-based). We believe that the sitting position has provided safe and efficient correction and facilitated primarily closed reduction. Moreover, bleeding control is easier in this position. In very severe deformities there is no other option to position the patient for spine surgery [10-11]. There are several drawbacks related to the sitting position: it may not be ergonomic for the surgeon, the anatomical relations are not that obvious, and cases of air embolism were described [10].

## Conclusions

The presented case shows that a spinal fracture in AS requiring surgical treatment may be also an opportunity to manage the pre-existing deformity. The strategy of closed reduction and deformity correction with subsequent three-column osteotomy alongside fracture fixation and fusion has proven efficient and safe. Positioning patients in the sitting position for the procedures was very helpful; it enabled efficient correction and avoided the difficulties of positioning a severely deformed and unstable spine.
